# Reconstruction of a Genome-Scale Metabolic Model for *Aspergillus oryzae* Engineered Strain: A Potent Computational Tool for Enhancing Cordycepin Production

**DOI:** 10.3390/ijms26146906

**Published:** 2025-07-18

**Authors:** Nachon Raethong, Sukanya Jeennor, Jutamas Anantayanon, Siwaporn Wannawilai, Wanwipa Vongsangnak, Kobkul Laoteng

**Affiliations:** 1 Institute of Nutrition, Mahidol University, Nakhon Pathom 73170, Thailand; nachon.rae@mahidol.ac.th; 2 Industrial Bioprocess Technology Research Team, Functional Ingredients and Food Innovation Research Group, National Center for Genetic Engineering and Biotechnology (BIOTEC), National Science and Technology Development Agency (NSTDA), Thailand Science Park, Pathum Thani 12120, Thailand; jutamas.ana@biotec.or.th (J.A.); siwaporn.wan@biotec.or.th (S.W.); kobkul@biotec.or.th (K.L.); 3 Department of Zoology, Faculty of Science, Kasetsart University, Bangkok 10900, Thailand; wanwipa.v@ku.ac.th; 4 Omics Center for Agriculture, Bioresources, Food, and Health, Kasetsart University (OmiKU), Bangkok 10900, Thailand

**Keywords:** *Aspergillus oryzae*, cordycepin, metabolic modeling, nutrient optimization, precision fermentation

## Abstract

Cordycepin, a bioactive adenosine analog, holds promise in pharmaceutical and health product development. However, large-scale production remains constrained by the limitations of natural producers, *Cordyceps* spp. Herein, we report the reconstruction of the first genome-scale metabolic model (GSMM) for a cordycepin-producing strain of recombinant *Aspergillus oryzae*. The model, *i*NR1684, incorporated 1684 genes and 1947 reactions with 93% gene-protein-reaction coverage, which was validated by the experimental biomass composition and growth rate. In silico analyses identified key gene amplification targets in the pentose phosphate and one-carbon metabolism pathways, indicating that folate metabolism is crucial for enhancing cordycepin production. Nutrient optimization simulations revealed that chitosan, D-glucosamine, and L-aspartate preferentially supported cordycepin biosynthesis. Additionally, a carbon-to-nitrogen ratio of 11.6:1 was identified and experimentally validated to maximize production, higher than that reported for *Cordyceps militaris*. These findings correspond to a faster growth rate, enhanced carbon assimilation, and broader substrate utilization by *A. oryzae*. This study demonstrates the significant role of GSMM in uncovering rational engineering strategies and provides a quantitative framework for precision fermentation, offering scalable and sustainable solutions for industrial cordycepin production.

## 1. Introduction

Cordycepin, a unique adenosine analog, exhibits various biological functions [1,2] due to its antineoplastic, anti-inflammatory, and immunomodulatory properties. Additionally, it reduces the symptoms of hyperlipidemia and diabetes [3], demonstrating significant potential as a pharmacological and health product. Cordycepin can be obtained via both chemical synthesis and biological methods [4]. Chemical synthesis is inappropriate for industrial production due to issues such as cost, efficiency, precursor availability, and environmental concerns [5,6]. Biological methods for cordycepin production mainly involve the use of native or engineered cordycepin-producing strains and artificial cultivation, including solid-state, submerged, or surface/static fermentation [7]. Currently, cordycepin is mainly derived from the fruiting bodies of *C. militaris*, which require complex environmental control. Furthermore, instability in the production process owing to strain degeneration during fruiting body cultivation has been observed with *C. militaris* [8]. Submerged fermentation of *C. militaris* has been intensively studied to overcome these limitations. Although its fermentation exhibits the highest cordycepin yield over other cordycepin-producing strains, the long production period leads to low cordycepin productivity (286.56–317.00 mg/L/d) [9]. Consequently, a practical production process for sustainable cordycepin production is desirable. A high cordycepin titer and cost-effective production process are required for commercial purposes.

With the availability of cordycepin biosynthetic genes, potent microbial cell factories, efficient genetic toolboxes, and the heterologous expression of cordycepin biosynthetic genes (*cns1* and *cns2*) under the control of inducible and constitutive promoters in yeasts (*Saccharomyces cerevisiae*, *Yarrowia lipolytica*, and *Komagataella phaffii*) and fungi (*Metarhizium robertsii* and *A. oryzae*) have been reported [10,11,12,13]. Among them, an engineered strain of *A. oryzae* offers the advantages of high cordycepin productivity (>550 mg/L/day), a short fermentation period (≤3 days), and the utilization of a broad substrate spectrum [13]. Compared to the engineered yeast strains (*Y. lipolytica* and *K. phaffii*) [11,12], *A. oryzae* is of great interest as a robust strain for large scale cordycepin production, in which it tolerates to extreme physical environments (pH and temperature) and capable utilize various carbon sources (C3, C5, C6, and C12 sugars) for growth and cordycepin production, whereas both yeast strains have limited xylose (C5) utilization. Xylose is an important renewable sugar and is considered a sustainable feedstock. In addition, extracellular cordycepin produced by *A. oryzae* requires only a simple cell separation step, facilitating the practical downstream process [13]. However, to utilize *A. oryzae* for cordycepin production at a commercial level, the development of a superior strain with high cordycepin yield and optimal cultivation processes for this filamentous fungus is required for economic feasibility.

In silico metabolic modeling is one of the most valuable tools for describing gene-protein-reaction (GPR) associations for all metabolic genes in an organism. This approach has been impressively successful in guiding metabolic engineering strategies and facilitating the study of biological systems [14]. A genome-scale metabolic model (GSMM) is a metabolic model reconstructed based on annotated genome sequences and detailed biochemical information. A GSMM provides a quantitative framework for stoichiometric biochemical models annotated with gene function coupled with mass-balance boundary conditions, enabling the simulation of how the metabolic network operates under different conditions. Several GSMMs have been developed and used to assess growth and metabolite production capabilities under various culture conditions and to identify metabolic engineering targets (overexpression or knockout) in silico. Many of these methods have been experimentally applied to enhance the production of the target products [15]. A GSMM was successfully used in cordycepin production to design an optimal culture medium for *C. militaris*. An approximately 3.5-fold increase in cordycepin yield was observed when the fungal cells were cultured in an in silico-designed medium (carbon-to-nitrogen [C:N] ratio of 8:1) [16]. For *A. oryzae*, a GSMM was reconstructed and simulated to describe the growth physiology of different carbon sources [17] and understand its secretory machinery [18] to enhance the production process of particular products. However, a GSMM of the cordycepin-producing *A. oryzae* strain is unavailable. Therefore, this study aimed to reconstruct a GSMM for the cordycepin-producing strain of *A. oryzae*. The constructed GSMM will be used to further rationalize cordycepin production. Precision fermentation guided by GSMM will enable the systematic identification of target gene amplification and optimization of nutrient supply for increasing cordycepin production. This systems-level approach will not only accelerate strain improvement strategies; it will also support the development of scalable, cost-effective, and sustainable bioprocesses for industrial cordycepin production.

## 2. Results

### 2.1. GSMM of the Cordycepin-Producing A. oryzae Strain

**Figure 1:**
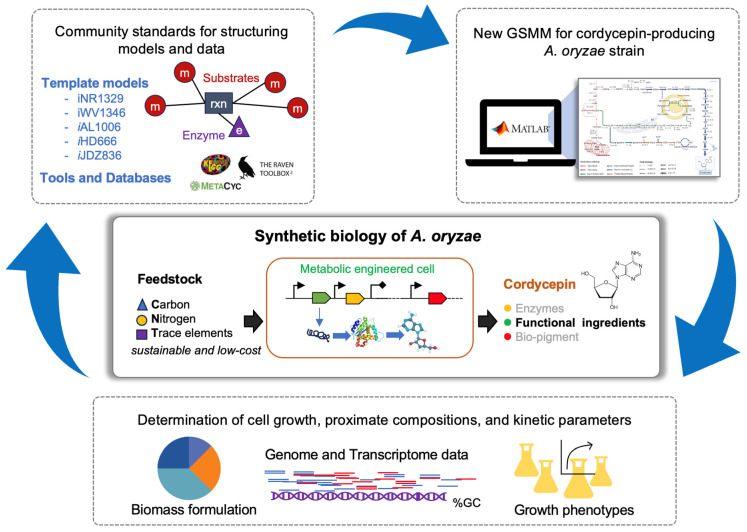
Schematic workflow for reconstructing a GSMM of an engineered *A. oryzae* strain. GSMM, genome-scale metabolic model.

A GSMM of the cordycepin-producing strain of *A. oryzae* was reconstructed based on the genomic and physiological information of *A. oryzae* BCC7051, which has previously been successfully engineered to produce cordycepin [13]. The reconstruction process commenced with a semi-automated approach using the GSMM of *C. militaris*, a native cordycepin-producing strain, as a template (Figure 1). This led to the creation of a draft metabolic network containing 1359 genes and 1685 reactions. Although this initiative provided a solid starting point, several reactions were missing (22% gaps). Thus, an additional draft metabolic network based on the GSMM of *A. oryzae* was developed, and 1386 genes and 966 biochemical reactions were identified through a homolog search against the *A. oryzae* RIB40 genome. When this new network was merged with the earlier network created from *C. militaris*, the total number of genes in the model increased to 1684 while reducing the gaps to 11%. These identified genes were categorized into three groups: genes that were orthologous to either *A. oryzae* RIB40 or *C. militaris* (353 and 336 genes, respectively), and those that were orthologous to both *A. oryzae* RIB40 and *C. militaris*, known as common orthologs (995 genes). These findings highlight the strain-specific metabolism of BCC7051, which is distinct from that of the RIB40 strain, and emphasize the close relationship between the metabolism of BCC7051 and *C. militaris*. Subsequently, 137 non-gene-associated spontaneous and exchange reactions were performed. Additionally, two reactions in the cordycepin biosynthetic pathway were incorporated into this draft without annotated genes.

The GSMM for the cordycepin-producing strain of *A. oryzae*, *i*NR1684, was reconstructed using 1684 genes and 1947 reactions associated with 1155 metabolites across four compartments (cytosol, mitochondria, peroxisome, and extracellular space). Compared with the GSMM characteristics of *C. militaris* CM01 and *A. oryzae* RIB40, the current GSMM (*i*NR1684) had the highest number of genes (Table 1). This suggests that the GSMM of the *A. oryzae* recombinant provides a complete metabolic network of the cordycepin-producing strain, potentially aiding in understanding metabolic processes and optimizing metabolic engineering strategies.

### 2.2. The GSMM Biomass Formulation

**Figure 2:**
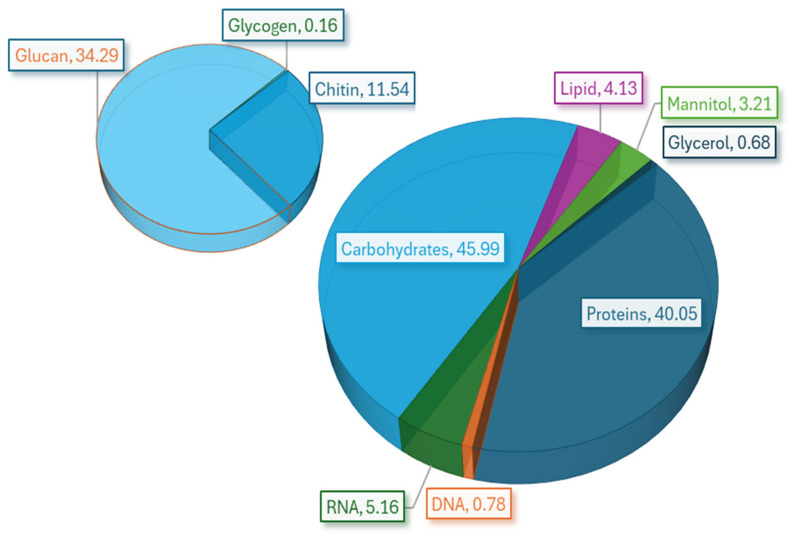
Biomass composition (% *w*/*w* DCW) of *A. oryzae* BCC7051. DCW, dry cell weight.

Accurately defining biomass is crucial for flux balance analysis (FBA) in growth behavior simulations. Thus, the overall stoichiometry of the metabolic precursors involved in the biomass synthesis reactions of *A. oryzae* BCC7051 was quantitatively assessed using both our experimental findings and relevant literature. The macronutrient composition of *A. oryzae* BCC7051 biomass, as shown in Figure 2, revealed that carbohydrates constituted 45.99% of the total composition, with glucans accounting for 34.29%. Proteins accounted for 40.05% of the total composition, while lipids accounted for 4.13%. Nucleic acids included 5.16% RNA and 0.78% DNA. Mannitol and glycerol comprised 3.21% and 0.68%, respectively. Chitin was present at 11.54%, and glycogen accounted for 0.16% of the total carbohydrate content. Overall, carbohydrates and proteins were predominant in the biomass of *A. oryzae* BCC7051, along with significant constituents of glucans and chitin (Figure 2). The proteomic analysis identified all 20 standard amino acids in varying proportions (% g/g dry cell weight [DCW]) in *A. oryzae* BCC7051, as summarized in Supplementary Table S1. L-Leucine (3.82%), L-arginine (3.27%), and L-glutamate (2.81%) were the most abundant amino acids, followed by L-serine, L-aspartate, and L-valine (2.72–2.33%). Lower levels of L-glycine, L-asparagine, and other amino acids (≤1.63%) were observed. Notably, although lipids comprised a small portion (4.13%), their composition in the biomass was nuanced. The lipid profile of *A. oryzae* BCC7051 biomass predominantly comprised triacylglycerol, ergosterol, and sterol esters, with smaller contributions from phospholipids and free fatty acids such as linoleic acid, stearic acid, oleic acid, and palmitic acid, as summarized in Table 2. This result highlights ergosterol and triacylglycerol as notable lipid components of *A. oryzae* BCC7051.

### 2.3. Scenario GSMM Validation by Optimizing Growth and Synthetic Cordycepin Production

GSMM validation of *i*NR1684 was conducted using two scenarios: one for the wild-type (WT) and the other for the cordycepin-producing strain. In the WT strain simulation, biomass was the objective function, and the flux to cordycepin production was set to zero, indicating no synthetic pathway for cordycepin. The cordycepin-producing strain also used biomass as the objective function. The production flux was adjusted based on experimental data, resulting in a functional synthetic pathway utilizing heterologous *cns*-encoding enzymes of *C. militaris* in *A. oryzae*. Simulation results were validated by comparing the growth rates of the two strains. Table 3 shows that the predicted growth rate agrees well with the experimental rate, with an error rate of less than 5% for both scenarios.

### 2.4. In Silico Identification of Gene Amplification Targets for Improving Cordycepin Production

Identifying key genes for deletion or amplification is a critical step in metabolic engineering to improve microbial strains for high production of desired bioproducts. This study employed flux scanning based on enforced objective flux (FSEOF) and FBA to identify the target genes that enhance cordycepin production in *A. oryzae*. For the FSEOF analysis, the cordycepin-producing strain was modeled under conditions that enforced cordycepin production, and cellular growth was set as the objective for optimization.

**Figure 3:**
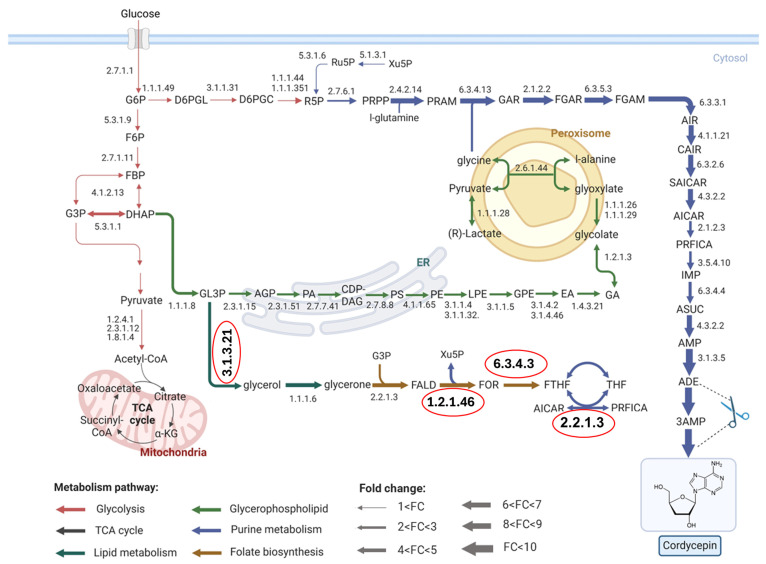
Highlighted fluxes relevant to cordycepin biosynthesis in *Aspergillus oryzae*. Red circles indicate gene amplification targets for the improvement of cordycepin production. Abbreviated metabolite names are as follows: G6P, alpha-d-glucose 6-phosphate; F6P, beta-d-fructofuranose 6-phosphate; FBP, D-fructose 1,6-bisphosphate; G3P, glyceraldehyde 3-phosphate; DHAP, glycerone phosphate; α-KG, α-ketoglutarate; D6PGL, 6-o-phosphono-d-glucono-1,5-lactone; D6PGC, 6-phospho-d-gluconate; R5P, ribose 5-phosphate; Ru5P, ribulose 5-phosphate; Xu5P, d-xylulose 5-phosphate; PRPP, 5-phospho-alpha-d-ribose 1-diphosphate; PRAM, 5-phospho-ribosylamine; GAR, n(1)-(5-phospho-d-ribosyl)glycinamide; FGAR, n(2)-formyl-n(1)-(5-phospho-d-ribosyl)glycinamide; FGAM, 2-formamido-n(1)-(5-phospho-d-ribosyl)acetamidine; AIR, 5-amino-1-(5-phospho-ribosyl) imidazole; CAIR, 1-(5-phospho-d-ribosyl)-5-amino-4-imidazolecarboxylate; SAICAR, (2S)-2-[5-amino-1-(5-phospho-β-D-ribosyl)imidazole-4-carboxamido]succinic acid; AICAR, 5-amino-1-(5-phospho-d-ribosyl)imidazole-4-carboxamide; PRFICA, 5-formamido-1-(5-phospho-d-ribosyl)imidazole-4-carboxamide; IMP, inosine monophosphate; ASUC, adenylosuccinate; AMP, adenosine-50-monophosphate; ADE, adenosine; 3AMP, adenosine-30-monophosphate; GL3P, glycerol 3-phosphate; AGP, acylglycerol 3-phosphate; PA, phosphatidate; CDP-DAG, CDP-diacylglycerol; PS, phosphatidylserine; PE, phosphatidylethanolamines; LPE, lysophosphatidylethanolamine; GPE, glycerol phosphatidylethanolamine; EA, Ethanolamine; GA, glycolaldehyde; FALD, formaldehyde; FOR, formate; FTHF, 10-formyltetrahydrofolate and THF, tetrahydrofolate.

The simulation results identified 20 metabolic reactions with Enzyme Commission (EC) numbers and GPR associations as potential amplification targets (slope > 1; Table S2). These reactions were computationally predicted to support enhanced cordycepin biosynthesis upon upregulation. FBA was used to simulate the metabolic responses of *A. oryzae* in two scenarios: (1) the WT and (2) cordycepin-producing strains. A comparative flux analysis revealed active fluxes relevant to cordycepin biosynthesis. The results revealed that 16 metabolic reactions with EC numbers and GPR associations involved in cordycepin production exhibited predominantly increased fluxes in the engineered strain (Log2FC > 2), as listed in Table S3. Regarding comparative flux analysis between the WT and cordycepin-producing strains, the identified active fluxes leading to cordycepin production in *A. oryzae* were mainly driven through the pentose phosphate pathway (PPP), which supplied the key precursors for purine biosynthesis (Figure 3). Interestingly, by integrating the reactions, EC numbers, and their associated GPRs from both the FSEOF and FBA simulations, four reactions with nine gene amplification targets for improving cordycepin production in *A. oryzae* were identified (Table 4). These included enzymes such as formate tetrahydrofolate ligase (EC 6.3.4.3), formaldehyde dehydrogenase (EC 1.2.1.46), and formaldehyde transketolase (EC 2.2.1.3), which collectively contribute to the generation of formylated tetrahydrofolate (formyl-THF) cofactors. These cofactors were essential for the conversion of 5-amino-1-(5-phospho-d-ribosyl)imidazole-4-carboxamide (AICAR) to 5-formamido-1-(5-phospho-d-ribosyl)imidazole-4-carboxamide (PRFICA) (Figure 3), highlighting folate metabolism as a critical regulatory node. Therefore, enhancing flux through folate biosynthesis may serve as a strategic metabolic engineering target to boost cordycepin production.

### 2.5. Optimizing Nutrients and the C:N Ratio Toward Rational Design of Synthetic Media for Cordycepin Overproduction in A. oryzae

**Figure 4:**
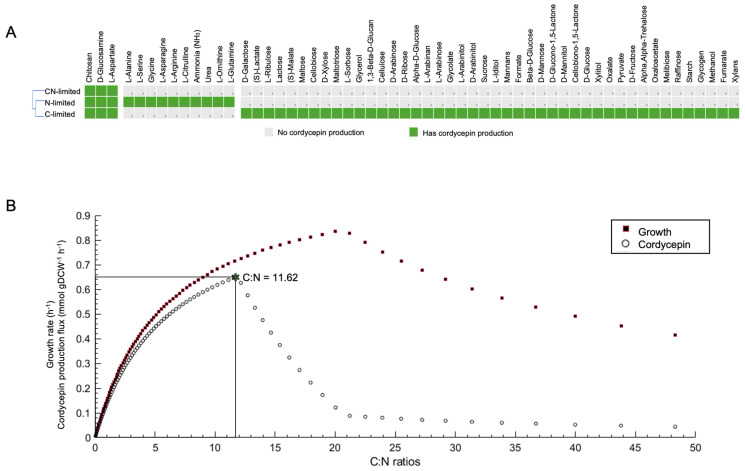
In silico identification of nutrients and carbon-to-nitrogen (C:N) ratios influencing cordycepin production in *Aspergillus oryzae*. (**A**) Cordycepin production fluxes are predicted below 57 single-nutrient supplementation scenarios. Simulations were performed using the *i*NR1684 model with growth fixed at 0.5 h^−1^. Each nutrient was added individually (1000 mmol) under three conditions: CN-limited (no glucose or ammonia), N-limited (1 mmol glucose), and C-limited (1 mmol ammonia). Only essential nutrients (oxygen, phosphate, sulfate) were constantly provided; (**B**) Effect of the C:N ratio on growth and cordycepin production. The optimal C:N ratio for balanced growth and cordycepin production was marked with a star.

Enhancing cordycepin production requires genetic modification and an optimized nutritional environment to support growth and metabolite synthesis. Although *A. oryzae* thrives on lignocellulosic biomass as a complex nutrient source, the specific nutrients that favor cordycepin biosynthesis remain largely unknown. Using the genome-scale model *i*NR1684, we explored the influence of individual nutrient uptake on cordycepin production. In total, 133 nutrient uptake reactions were assessed under three physiological conditions to simulate carbon and/or nitrogen limitations. In silico analysis showed that the cordycepin production fluxes were affected by supplementation with 57 nutrients (Table S4), as illustrated in Figure 4A. Among these, three nutrients—chitosan, D-glucosamine, and L-aspartate—were found to support cordycepin production by *A. oryzae,* even in the absence of glucose and ammonia. Under nitrogen-limited conditions (with glucose but no ammonia), 11 nutrients could serve as nitrogen sources to support cordycepin biosynthesis. However, the cordycepin yield was dependent on the availability of carbon (glucose). Conversely, under carbon-limited conditions (with ammonia supplied but no glucose), 44 nutrients acted as carbon sources; however, the cordycepin yield was similarly dependent on nitrogen (ammonia) availability. These findings suggest that *A. oryzae* utilizes a wide range of nutrients, particularly carbon sources, for cordycepin production, but optimal output requires a balanced supply of carbon and nitrogen. To address this issue, the model was used to optimize the C:N ratio, and as shown in Figure 4B, the optimal C:N ratio for cordycepin production in *A. oryzae* was identified as 11.6:1. Experimental validation by cultivating *A. oryzae* in a series of synthetic media different in C:N ratio (Supplementary Table S5) proved that the predicted C:N ratio is precise. As shown in Table 5, the highest cordycepin production was observed in culture C:N ratio 11.6:1 (1.521 gL^−1^), which was higher than that of the lowest C:N ratio (7.3:1) and the highest C:N ratio (58.2:1) by about 1.2 and 1.6-fold, respectively (Table 5).

## 3. Discussion

To develop an efficient cordycepin production process with economic feasibility, identifying or engineering a microbial strain that has robust metabolic flux toward the high target product is one important step. In addition, cost-effective production processes (upstream and downstream) that are high production yield, simple, and can be scaled up are critical processes. With the increase in omics and experimental data, in silico metabolic modeling has emerged as a transformative tool for uncovering gene-function relationships and enabling precision strain engineering. GSMMs have revolutionized the design of microbial cell factories, achieving remarkable improvements in the production of various metabolites, including cordycepin [14,16,17,18]. Significantly, a 3.5-fold increase in cordycepin yield in *C. militaris* was obtained through model-guided medium optimization [16]. Although a GSMM has been developed for *A*. *oryzae* [17], to date, it has not been tailored to cordycepin-producing strains. To our knowledge, this is the first report of a GSMM of an engineered *A. oryzae* strain for cordycepin production. The resulting model, *i*NR1684, was systematically reconstructed using genomic and physiological data from *A. oryzae* BCC7051 [13,19]. Compared to *A. oryzae* BCC7051, RIB40 [17], and *C. militaris* CM01 [16], *i*NR1684 includes more metabolic genes and 93% GPR-supported reactions, focusing on cordycepin pathways. *i*NR1684 was validated by incorporating an experimentally derived biomass composition and accurately predicting growth rates within 5% of the experimental values under WT and cordycepin-producing conditions.

The gene amplification targets essential for increasing cordycepin production were systematically identified through 20 candidate reactions from FSEOF and 16 flux-increased reactions from FBA, predominantly involving the PPP. The integration of these results revealed four key metabolic reactions linked to nine gene amplification targets (Table 4). Interestingly, a group of genes (formate tetrahydrofolate ligase, formaldehyde dehydrogenase, and formaldehyde transketolase) involved in folate biosynthesis were predicted to be critical for purine biosynthesis and cordycepin formation in the *A. oryzae* engineered strain. The identification of folate metabolism as a regulatory bottleneck suggests that enhancing the flux towards formyl-THF production could be a promising strategy for augmenting cordycepin biosynthesis. Formyl-THF acts as a one-carbon donor in the purine biosynthetic pathway, facilitating the formation of key intermediates, such as PRFICA [20]. Previous studies have emphasized that an ample supply of formyl-THF is crucial for efficient nucleotide and nucleotide analog syntheses [21]. Supporting this, the recent engineering of *K. phaffii* for methanol-based cordycepin biosynthesis demonstrated that introducing cordycepin pathway genes drives global metabolic and transcriptional reprogramming, particularly enriching the one-carbon metabolism and purine biosynthesis pathways [12]. Moreover, a comprehensive investigation of folate biosynthesis genes in relation to cordycepin production requires further study. This can suggest the cellular response to targeted manipulations of folate pathway genes, such as gene overexpression and knockout experiments, and the downstream effects on nucleotide biosynthesis, e.g., purine precursors. Additionally, the functional roles of other candidate proteins beyond *Cns1* and *Cns2* are needed for further exploration, particularly in relation to the influence on central carbon metabolism and the redistribution of metabolic flux towards cordycepin production.

To translate the in silico findings into actionable strategies for developing the fermentation process, the model was further utilized to optimize the nutrient composition and C:N ratio toward the rational design of a synthetic medium for cordycepin overproduction in *A. oryzae*. By systematically analyzing nutrient uptake profiles and simulating metabolic responses under varied nutrient availabilities, chitosan, D-glucosamine, and L-aspartate were identified as the preferable nutrients for enhancing cordycepin production in the *A. oryzae* engineered strain. This result contrasts with *C. militaris*, which grows naturally in insect hosts but cannot utilize chitosan as a nutrient source [16]. In contrast, *A. oryzae* BCC7051 was found to possess chitosanase (EC 3.2.1.132), an enzyme capable of degrading chitosan into chitooligosaccharides primarily composed of d-glucosamine, with occasional incorporation of N-acetylglucosamine [22]. The identification of chitosan as a supportive nutrient for cordycepin production offers new opportunities for rational media optimization. As a biopolymer rich in carbon and nitrogen, chitosan can simultaneously fulfill the metabolic demands required for biomass accumulation and secondary metabolite synthesis [23]. Additionally, chitosan degradation by chitosanase activity in *A. oryzae* generates D-glucosamine, a readily assimilable substrate, as shown in this study, supporting cordycepin biosynthesis even without conventional carbon and nitrogen sources. Using chitosan, a sustainable and low-cost byproduct of seafood processing, aligns with circular bioeconomy principles and can enhance the cost-effectiveness and environmental sustainability of industrial cordycepin production processes [24]. In addition to these specific nutrients, BCC7051 utilizes a wide range of carbon and nitrogen sources for cordycepin biosynthesis. However, optimal production depends on a balanced supply of carbon and nitrogen. Accordingly, the model was used to optimize the C:N ratio, with simulations predicting an optimal value of 11.6:1 for maximizing cordycepin production in *A. oryzae*. Interestingly, in silico C:N ratio prediction was supported by experimental validation (Table 5). In addition, this prediction also aligns with the C:N ratio (11.1:1) of the optimal culture medium obtained from the Design of Experiments analysis for cordycepin production by the engineered *A. oryzae* strain [25]. This finding highlights the importance of balancing growth-associated metabolism and secondary metabolite biosynthesis because excess nitrogen (low C:N ratio) may repress secondary metabolism, whereas insufficient nitrogen limits biomass accumulation. This predicted C:N ratio was considerably higher than that reported for *C. militaris*, where the optimal ratios typically range between 5:1 and 8:1 [16,26]. The higher C:N requirement observed in *A. oryzae* is consistent with its faster growth rate, more robust carbon assimilation capacity, and broader substrate utilization spectrum than those of *C. militaris*. Previous studies have demonstrated that *A. oryzae* exhibits efficient nitrogen metabolism supported by high-affinity ammonium transporters. This facilitates rapid biomass accumulation and demands a proportional increase in carbon flux to sustain growth and metabolite biosynthesis [27]. Additionally, its broad enzymatic repertoire, including amylases, proteases, and lipases, enables the degradation of complex substrates, such as starch, proteins, and lipids, allowing the organism to adapt to various cultivation conditions. These features collectively contribute to the fungi’s ability to thrive in carbon-rich environments and support high levels of cordycepin production under diverse nutrient settings, thus justifying its higher optimal C:N ratio compared to that of more metabolically specialized fungi, such as *C. militaris*. These insights highlight the species-specific metabolic demands and provide a quantitative framework for designing synthetic media tailored to the specific metabolic characteristics of recombinant *A. oryzae*. Thus, fine-tuning the C:N ratio to 11.6:1 may represent a practical and scalable strategy to enhance cordycepin yield in industrial fermentation settings.

In conclusion, this study demonstrates the power of metabolic modeling in revealing species-specific metabolic demands and guiding rational strain and medium engineering. The *i*NR1684 model provides a quantitative framework for precision fermentation of cordycepin in *A. oryzae* and opens new avenues for yield enhancement through targeted metabolic interventions and media formulation. Fine-tuning the one-carbon metabolism pathway and optimizing the C:N ratio offer practical and scalable strategies to support the industrial production of cordycepin. However, as with most GSMMs, *i*NR1684 is limited by the absence of regulatory and kinetic constraints. Integration of multi-omics data, including transcriptomics, proteomics, and metabolomics, that is essential for capturing condition-specific regulatory and metabolic states [28,29] proposed to be further studied in the *A. oryzae* model. The availability of its genetic toolbox and gene targeting system will facilitate experimental validation via gene knockout or overexpression, which are critical steps to further validate in silico predictions and to guide rational and effective metabolic engineering strategies for *A. oryzae*.

## 4. Materials and Methods

### 4.1. Fungal Strain and Cultivation

WT and recombinant strains (AoCordy-T1) of *A. oryzae* [13] were used in this study. For inoculum preparation, the fungal cells were grown on polished rice at 30 °C for 5 days and suspended spores in 0.01% (*v*/*v*) Tween 80 solution. To acquire data for GSMM reconstruction, fungal spores at a final concentration of 10^6^ spores/mL were inoculated into a synthetic medium with 4% (*w*/*v*) glucose and 0.23% (*w*/*v*) NH_4_Cl as the nitrogen source, modified from a study by Jeennor et al. [13]. The cultures were incubated at 30 °C on a rotary shaker at 200 rpm for 4 days.

### 4.2. Determination of Cell Growth, Proximate Compositions, and Cordycepin Production of A. oryzae Strains

The fungal cultures were collected at different times to measure the DCW, residual sugar, proximate composition, and cordycepin concentration. To quantify the DCW, mycelial cells were harvested by filtration using Miracloth (EMD Chemicals, Gibbstown, NJ, USA) and dried at 60 °C in a hot air oven (Memmert, Schwabach, Germany) until a constant weight was obtained. High-pressure liquid chromatography (HPLC, Ultimate 3000, Thermo, Waltham, MA, USA) with a refractive index detector (RID) and an Aminex HPX-87H ion exclusion column (Bio-Rad Laboratories, Hercules, CA, USA) was used to measure the amount of residual sugars in the fermented broths of the fungal cultures. The HPLC conditions and calibration curves of the glucose standard were set as previously described [13]. To obtain the proximate composition (carbohydrates, fibers, proteins, lipids, ash, and other elements) of *A. oryzae*, the dried mycelia of *A. oryzae* collected from the exponential growth phase (48 h) were analyzed for proximate composition using a service of Central Laboratory Co., Ltd. (Bangkok, Thailand).

The concentration of cordycepin in the fermented broth of the *A. oryzae* recombinant strain was measured using an HPLC equipped with a diode array detector and a C18 column (AcclaimTM 120; 5 μm, 4.6 mm × 150 mm). The HPLC conditions were set in compliance with a published methodology [30]. The cordycepin compound was identified by comparing its retention times with those of an authentic standard (Sigma-Aldrich, Saint Louis, MO, USA) using UV spectra (260 nm). A standard curve correlating the area with known substance concentrations was used to quantify cordycepin concentration. The kinetic parameters for mycelial growth and cordycepin production were determined.

### 4.3. Reconstruction of GSMM for Cordycepin-Producing A. oryzae Strain

The GSMM for cordycepin-producing *A. oryzae* strains was developed through reference-based reconstruction using the Reconstruction, Analysis, and Visualization of Metabolic Networks (RAVEN) toolbox [31]. This process leverages the protein orthology and preexisting metabolic data to establish a functional model. The first step involved the acquisition of a reference model from *C. militaris* (*i*NR1329), which provided key GPR associations relevant to cordycepin biosynthesis [16]. Orthologous proteins were then identified between *A. oryzae* BCC7051 and the reference model *i*NR1329. This was accomplished through a bi-directional sequence alignment using the getBlast function, with specific parameters (E-value cut-off of 10^−50^, identity ≥ 40%, and alignment length ≥ 200 amino acids) to identify the orthologs. A draft model of *A. oryzae* BCC7051 was created using the getModelFromHomology function. In addition to orthologous protein reactions, non-gene-associated (NGA) reactions, such as spontaneous, transport, and exchange reactions, were incorporated to capture the metabolic capabilities of the model. NGA reactions from the reference model were excluded from the draft model to enhance completeness, including specific reactions involved in cordycepin biosynthesis. Next, the reconstructed model was improved by incorporating an earlier GSMM for *A. oryzae* (*i*WV1346) [17]. A metabolic network was generated using protein orthology inference between *A. oryzae* BCC7051 and *i*WV1346. The draft model was merged with the metabolic network. To prevent the duplication of metabolites and reactions, identifiers were standardized based on databases such as MetaCyc [32] and Kyoto Encyclopedia of Genes and Genomes [33]. The grRules of the model were also standardized after merging. For growth simulation, biomass reactions specific to *A. oryzae* BCC7051 were introduced into the merged model, and FBA was used to optimize the feasibility of these biomass reactions. Reactions causing simulation errors, including duplicates, were eliminated. Finally, alternative homolog networks from *Penicillium chrysogenum* (*i*AL1006) [34], *Aspergillus nidulans* (*i*HD666) [35], and *Neurospora crassa* (*i*JDZ836) [36] were used for gap filling. This was achieved by mapping EC numbers and orthologous proteins to enhance the overall coverage of the model.

### 4.4. Formulation of Biomass Reactions

The cell biomass and proximate compositions obtained in this study, as well as the compositions of amino acids, monosaccharides, free fatty acids, sphingolipids, and sterol esters obtained from previous reports [37,38,39], were collected to formulate the biomass reactions. The weight of each nucleotide (DNA and RNA) in *A. oryzae* BCC7051 was calculated using the reported guanine-cytosine contents of 47% and 51% [19], respectively. Reactions involving the biomass composition of *A. oryzae* BCC7051 were also included. The accuracy and reliability of the results were then ensured through manual curation, which involved conducting experimental measurements, analyzing biochemical textbooks, and reviewing the relevant literature.

### 4.5. GSMM Simulation, Validation, and Analysis

The objective function was set as biomass production to maximize the growth predictions under various constraints using FBA. The adenosine triphosphate (ATP) maintenance reaction (m_ATP_) was restricted to 1 mmol gDW^−1^ h^−1^, and the oxidative phosphorylation efficiency (P/O ratio) was fixed at 2.5 based on fungal models from previous studies [16,17]. To optimize Y_xATP_ for the biomass synthesis of *A. oryzae* BCC7051, the maximum specific growth rate (μ_max_) obtained from the glucose cultivation was used in the ATP cost adjustment through FBA. Before its application in physiological growth analysis via FBA, the GSMM underwent growth parameter fitting to ensure optimal performance. The glucose uptake rate was set according to the experiment. Small-molecule metabolites, such as CO_2_, H_2_O, SO_3_, NH_3_, PO_4_, and O_2_, were allowed to be freely transported across the cell membrane. The simulation was divided into two scenarios: (1) the WT and (2) the cordycepin-producing strains. For the WT strain, intracellular metabolic fluxes were calculated using biomass as the objective function, and the flux for cordycepin production was constrained to zero. Simulation of the WT strain resulted in a scenario where there appeared to be no synthetic pathway for cordycepin. For the cordycepin-producing strain, biomass was designated as the objective function, and the flux for cordycepin production was adjusted based on experimental data. This simulation of the cordycepin-producing strain resulted in a scenario where a synthetic pathway utilizing heterologous *cns*-encoding enzymes for the production of cordycepin, sourced from *C. militaris*, was effectively constructed and functional in *A. oryzae* BCC7051. The GSMM simulation resulting from scenarios 1 and 2 was validated by comparing the growth rates of the WT and cordycepin-producing strains. Additionally, to explore the potential metabolic route for cordycepin biosynthesis in the cordycepin-producing *A. oryzae* strain, the following changed function was applied, with a cut-off variation of 95% between scenarios 1 and 2, to pinpoint the active fluxes pertinent to cordycepin production. In parallel, the model constrained by the cordycepin-producing scenario was employed for FSEOF using the FSEOF function in the RAVEN toolbox, with both growth (bmOUT) and cordycepin production (cordycepinOUT) set as the target reactions.

To evaluate the effect of individual nutrient uptake on cordycepin production, the model was tested using 133 nutrient uptake reactions under three distinct conditions, with modifications adapted from a previous study [16]. These conditions included (i) carbon- and nitrogen-limited (CN-limited)—only essential nutrients (oxygen, phosphate, and sulfate) provided at 1000 mmol, with no glucose or ammonia available; (ii) nitrogen-limited (N-limited)—essential nutrients plus 1 mmol of glucose; and (iii) carbon-limited (C-limited)—essential nutrients plus 1 mmol of ammonia. In each case, other nutrients were individually tested by supplying them one at a time at an upper limit of 1000 mmol to determine their impact on cordycepin flux. The target objective function was set to cordycepin production flux (cordycepinOUT), and the growth rate (bmOUT) was fixed at 0.5 h^−1^ across all simulations. The C:N ratio was optimized using the POPCORN function, following the approach described by Raethong et al. [16]. Finally, the in silico predicted C:N ratio was experimentally validated using a series of synthetic media (C:N ratios = 7.3:1 to 58.2:1) as shown in Supplementary Table S5. The cultures were incubated at 30 °C on a rotary shaker at 200 rpm for 2 days.

### 4.6. Data Analysis

All data presented are the mean values of three independent experiments. Statistical data analysis was performed using Duncan’s multiple range test in the Statistical Package for the Social Sciences (SPSS) 11.5 program for Windows, and the data were considered statistically significant at *p* < 0.01.

## Figures and Tables

**Table 1:** Comparative characteristics of genomes and GSMMs of *A. oryzae* BCC7051 and related strains.

Genomic Characteristics	*A. oryzae* BCC7051	*A. oryzae* RIB40	*C. militaris* CM01
Genome size (Mb)	38.51	37.20	32.20
No. of protein-coding genes	11,456	12,096	9651
GSMM characteristics	*i*NR1684 (This study)	*i*WV1346	*i*NR1329
Total genes	1684	1346	1329
Total metabolites	1155	810	1171
Total reactions	1947	2360	1821
- GPR associations (% gap)	1810 (11)	2174 (9)	1684 (14)
- Non-GPR associations	137	186	137
Compartments	4	4	4

GSMM, genome-scale metabolic model; GPR, gene-protein-reaction.

**Table 2:** Stoichiometric coefficients of biomass components in *i*NR1684.

Biomass Component	Average Molecular Weight (g/mol)	Content (g/100 gDCW)	Stoichiometric Coefficient (mmol/gDCW)
Proteins	127.03	40.05	3.153
Carbohydrates	-	-	-
Chitin	203.20	11.54	0.568
Glucan	162.10	34.29	2.115
Glycogen	666.60	0.16	0.002
RNA	495.30	5.16	0.104
DNA	482.73	0.78	0.016
Lipids	-	-	-
Triacylglycerol	821.95	1.28	0.016
Phosphatidylcholine	744.76	0.15	0.002
Phosphatidylethanolamine	701.67	0.06	0.001
Palmitic acid (16:0)	200.32	0.17	0.009
Oleic acid (18:1 *n*-9)	242.40	0.15	0.006
Stearic acid (18:0)	239.20	0.33	0.014
Linoleic acid (18:2 *n*-6)	254.41	0.63	0.025
Arachidic acid (20:0)	265.30	0.01	0.0003
Sterol esters	302.45	0.48	0.016
Ergosterol	396.65	0.87	0.022
Others	-	-	-
D-Mannitol	182.20	3.21	0.176
Glycerol	92.10	0.68	0.074

DCW, dry cell weight.

**Table 3:** Kinetic parameters of the *A. oryzae* strains using glucose as a carbon source.

Parameters	Wild Type	Cordycepin-Producing Strain
Exponential phase (h)	24–48	24–48
Growth rate, µ_max_ (h^−1^)	0.032 ± 0.004	0.025 ± 0.002
Biomass production (gDW L^−1^)	8.690 ± 0.070	5.860 ± 0.170
Sugar uptake rate (mmol gDW^−1^ h^−1^)	0.632 ± 0.042	0.549 ± 0.026
Cordycepin production rate (mmol gDW^−1^ h^−1^)	-	0.013 ± 0.001
Cordycepin yield on biomass (mg gDW^−1^)	-	81.975 ± 0.005
Cordycepin titer (mg L^−1^)	-	479.970 ± 13.590
In silico growth rate (h^−1^)	0.032	0.025
% error rate	0.94%	2.77%

**Table 4:** List of gene amplification targets for improving cordycepin production.

EC Number	Enzyme Name	GPR Association
1.2.1.46	Formaldehyde dehydrogenase	OAory_01014160 OAory_01017500 OAory_01018710
2.2.1.3	Formaldehyde transketolase	OAory_01025450 OAory_01077260 OAory_01105810
6.3.4.3	Formate tetrahydrofolate ligase	OAory_01002190
3.1.3.21	Glycerol-3-phosphate phosphohydrolase	OAory_01027100 OAory_01104250

EC, Enzyme Commission.

**Table 5:** Experimental validation of the in silico prediction of optimal C:N ratios for growth and cordycepin production in *A. oryzae*.

Production Data	C:N Ratio						
	7.3:1	9.7:1	11.6:1	14.6:1	19.4:1	29.1:1	58.2:1
Cell biomass (DCW, gL^−1^)	20.315 ± 0.276	19.544 ± 0.079	18.019 ± 0.143	16.635 ± 1.557	14.097 ± 0.683	12.373 ± 0.564	9.619 ± 0.284
Biomass productivity (gL^−1^ h^−1^)	0.423 ± 0.006	0.407 ± 0.002	0.375 ± 0.003	0.347 ± 0.032	0.294 ± 0.014	0.258 ± 0.012	0.2 ± 0.006
Cordycepin titer (gL^−1^)	1.288 ± 0.017	1.366 ± 0.01	1.521 ± 0.015	1.416 ± 0.025	1.35 ± 0.003	1.133 ± 0.008	0.95 ± 0.003
Cordycepin productivity (gL^−1^ h^−1^)	0.027 ± 0	0.028 ± 0	0.032 ± 0	0.029 ± 0.001	0.028 ± 0	0.024 ± 0	0.02 ± 0

## Data Availability

Codes and models developed and used in this study are available at https://github.com/nachonase/corOryzae (accessed on 15 July 2025).

## Supplementary Materials

The following supporting information can be downloaded at: https://www.mdpi.com/article/10.3390/ijms26146906/s1.

## Abbreviations

The following abbreviations are used in this manuscript:

AICAR	5-amino-1-(5-phospho-d-ribosyl)imidazole-4-carboxamide
DCW	dry cell weight
EC	enzyme commission
FBA	flux balance analysis
FSEOF	flux scanning based on enforced objective flux
GPR	gene-protein-reaction
GSMM	genome-scale metabolic model
PPP	pentose phosphate pathway
PRFICA	5-formamido-1-(5-phospho-d-ribosyl)imidazole-4-carboxamide
